# Therapeutic effects of ranibizumab in patients with polypoidal choroidal vasculopathy

**DOI:** 10.1186/s12886-019-1156-4

**Published:** 2019-07-19

**Authors:** Xiaoya Gu, Xiaobing Yu, Hong Dai

**Affiliations:** Department of Ophthalmology, Beijing Hospital, National Center of Gerontology, No.1 Dahua Road, Dongdan, Dongcheng District, Beijing, China

**Keywords:** Anti-VEGF, Photodynamic therapy, Polypoidal choroidal vasculopathy

## Abstract

**Background:**

There is no consensus on the optimal initial treatment for polypoidal choroidal vasculopathy (PCV). Our study aimed to report the efficacy of repeated injections of intravitreal ranibizumab with or without photodynamic therapy for the treatment of PCV and to determine the possible factors predictive of visual outcomes.

**Methods:**

The results of the initial treatment of 40 patients with PCV with 3 monthly injections of ranibizumab were retrospectively reviewed. We compared the results in terms of the best corrected visual acuity (BCVA), the central retinal thickness (CRT), the number of injections, the regression rates of polyps and the rates of the reduction of subretinal fluid.

**Results:**

At the 3-month follow-up, the mean BCVA was significantly increased by 7.3 ± 12.4 letters compared to baseline (*p* < 0.01). At the 12-month follow-up, the mean BCVA was increased by 3.4 ± 15.4 letters compared to baseline, and there was no significant difference (*p* > 0.05). The mean CRT at the 12-month follow-up was 593.58 ± 243.64 μm, with an average decrease of 101.55 ± 256.07 μm compared to baseline (*p* < 0.01). Fifteen eyes (18.8%) showed the complete regression of polyps, and 22 eyes (27.5%) showed a reduction in polyps. The baseline VA, the reduction in subretinal fluids and the greatest lesion diameter were significant independent factors that were predictive of improved VA at the final follow-up.

**Conclusions:**

Three monthly injections of ranibizumab as an initial treatment could significantly improve VA in PCV patients in the short term. At 12 months postinjection, ranibizumab treatment could stabilize VA in most PCV patients. The baseline VA, the reduction in subretinal fluids and the greatest lesion diameter were predictive factors for the relative improvement of VA at the final follow-up.

## Background

Polypoidal choroidal vasculopathy (PCV) was first defined by Yannuzzi in 1982. It is characterized by the presence of orange-red polypoidal vascular lesions beneath the retinal pigment epithelium (RPE) that are usually associated with hemorrhagic or serous pigment epithelial detachment (PED) [1, 2]. The gold standard for PCV diagnosis is indocyanine green angiography (ICGA). It is defined as the presence of grape-like clusters of hyperfluorescence within the choroidal circulation within the first 6 min after the injection of indocyanine green dye, with or without an associated branching vascular network (BVN) [3]. PCV accounts for 10–54% of previously diagnosed age-related macular degeneration (AMD) cases in Asian populations [4, 5]. The presence of hemorrhagic or serous PED in PCV may lead to recurrent episodes of vitreal hemorrhage and exudative maculopathy, which may cause significant and permanent vision loss and therefore seriously affect quality of life [6].

Among the many treatment options, photodynamic therapy (PDT) has been proposed as a standard treatment, especially for lesions under the fovea. Studies have shown that PDT could lead to the complete regression of polyps in 73–99% of patients with stable visual acuity [7–9]. However, for lesions with multiple, widespread polyps, PDT could damage normal tissue, and repeated PDT could cause subsequent hemorrhage. Recently, studies have shown that samples of aqueous humor and tissue from PCV patients show increased levels of vascular endothelial growth factor (VEGF) [10, 11]. This indicated another treatment modality that could be used for PCV – anti-VEGF agents. Theoretically, ranibizumab could lead to the resolution of subretinal hemorrhage and decrease in macular edema. To achieve synergistic treatment effects, PDT and anti-VEGF injections were combined. There are studies that report that combined therapy led to improved VA, a reduced subretinal hemorrhage rate and an increased lesion regression rate compared to PDT alone [12–14]. However, the use of ranibizumab monotherapy to treat PCV has not been sufficiently explored. Two randomized clinical trials (EVEREST and LAPTOP) reported that ranibizumab was more effective in improving VA than PDT [14, 15]. Several studies have also reported the stabilization or even the improvement of vision in PCV patients who received anti-VEGF treatment alone [14–20]. There is currently no consensus on the optimal treatment plan in terms of the use of combination therapy or anti-VEGF therapy for the initial treatment of PCV. The aim of our study is to report the efficacy of repeated injections of intravitreal ranibizumab with or without PDT for the treatment of PCV and to determine the possible factors predictive of visual outcomes.

## Methods

### Study population

This was a retrospective study conducted in the Department of Ophthalmology at Beijing Hospital. Patients were recruited and data were collected between February 2012 and May 2016. Seventy-four treatment-naïve patients with PCV were enrolled in the study, and data was collected for a total of eighty eyes. Written informed consent was obtained from all patients. To confirm the diagnosis, all patients underwent a comprehensive examination, including slit lamp examination, optical coherence tomography (OCT), fluorescein angiography (FA), indocyanine green angiography (ICGA) and fundus photography. The diagnosis of PCV was confirmed by the presence of single or multiple subretinal focal hyperfluorescences within 6 min after the injection of indocyanine green dye during ICGA, with or without an associated choroidal interconnecting vascular network. The complete regression of polyps was defined as the disappearance of previous focal hyperfluorescence. The best corrected visual acuity (BCVA) was measured with the Early Treatment Diabetic Retinopathy Study (ETDRS) chart. The central retinal thickness (CRT) was measured with OCT. The greatest lesion diameter was determined by measuring the longest diameter of the largest polypoidal lesion in ICGA images obtained during the early and late phases. Patients were excluded from participation if they 1) received previous PCV treatment, including anti-VEGF injections, PDT and focal laser photocoagulation, 2) had a history of other ocular diseases that could damage visual acuity, including but not limited to age-related macular degeneration, pathological myopia and cataracts, and 3) had undergone intraocular surgery (except uncomplicated cataract extraction with intraocular lens implantation). All patients were scheduled to receive three monthly intravitreal injections of ranibizumab (Lucentis, 0.5 mg). Patients with a decrease in the BCVA ≥5 letters compared to that obtained during previous visits or the presence of persistent/recurrent fluid during OCT after 3 months were subject to additional treatments as needed, including intravitreal injections of ranibizumab and PD,. The study was carried out in accordance with the Declaration of Helsinki.

### Intravitreal injection technique

Before the operation was performed, 3 days of topical ofloxacin was administered according to the Chinese standard operating process for intravitreal injection [13, 21]. Injections were administered in sterile conditions in an operating room. Ofloxacin was injected into the vitreous 3.5–4.0 mm posterior to the limbus. The patient was instructed to use topical ofloxacin for 3 days following the injection to avoid infection.

### Outcome measures

Follow-up visits were scheduled every month after the initial injection. During each visit, the BCVA and the intraocular pressure (IOP) were recorded, and OCT was performed. FA and ICGA were performed at 12 months postinjection or earlier, if necessary, to confirm the regression of polyps. The outcome measures were defined as the changes in BCVA and CRT at the 3-month follow-up and the last follow-up, the regression rate of polyps, the reduction rate of subretinal fluid at the last follow-up and the number of injections.

### Statistical analysis

All data were processed using IBM SPSS software version 19 (IBM Statistics, Inc., Chicago, IL, USA). Descriptive statistics were represented as the mean and the standard deviation. A paired Student’s t-test was used to compare the BCVA and the CRT obtained at baseline and at each follow-up. To determine the possible factors predictive of the BCVA at the 12-month follow-up, simple linear regression analysis was used. Subsequently, multivariate linear regression analysis was used to determine the independent factors. The statistical differences were defined as *p* < 0.05.

### Trial registration

ChiCTR1900021656: date of registration: 3/3/2019, retrospectively registered.

## Results

### Patient characteristics

The detailed baseline characteristics of the patients are listed in Table 1. The gender distribution consisted of 45 men (60.8%) and 29 women (39.2%). The patients’ ages ranged from 48 to 85, with a mean age of 68.9 ± 9.3 years. Of the 80 eyes for which data was collected, 69 were phakic and 11 were pseudophakic due to cataract surgery performed at least 6 months prior to the study. At baseline, the mean BCVA was 49.7 ± 18.0 letters, and the mean CRT was 695.1 ± 319.5 μm. Seventy eyes (87.5%) showed PED, and 71 eyes (88.8%) showed the presence of associated subretinal fluids by OCT. Twenty-five eyes (31.3%) showed PCV lesions associated with retinal or subretinal hemorrhage. All eyes had not been treated previously for PCV.

**Table 1:** Patient characteristics at baseline

	Number	Percentage
Eyes/Patients	80/74	
Gender (male)	45	60.8%
Age (years)	68.9 ± 9.3	
Lens status (phakic)	69	86.3%
Mean baseline BCVA (letters)	49.7 ± 18.0	
Mean baseline CRT (μm)	695.1 ± 319.5	
PED	70	87.5%
Subretinal fluids	71	88.8%
Retinal/subretinal hemorrhage	25	31.3%

*BCVA* best corrected visual acuity, *CRT* central retinal thickness, *PED* pigment epithelial detachment

### Additional treatments

All treatments are listed in Table 2. Fifty-eight (72.5%) eyes with persistent or recurrent hemorrhagic/exudative maculopathy shown by OCT after 3 months were subject to additional treatments. Forty eyes (50%) were subject to repeated intravitreal injections of ranibizumab, 9 eyes (11.3%) received PDT and 9 eyes (11.3%) received combination treatment with ranibizumab and PDT. The mean number of ranibizumab injections was 4.46 ± 1.63, and the range was from 3 to 9.

**Table 2:** Treatment of 80 eyes diagnosed with PCV

Number of eyes	Treatment
22	Ranibizumab× 3
40	Ranibizumab× 3 + Ranibizumab×(1~6)
9	Ranibizumab× 3 + PDT × 1
9	Ranibizumab× 3 + PDT × 1 + Ranibizumab×(1~5)

*PCV* polypoidal choroidal vasculopathy, *PDT* photodynamic therapy

### Visual outcomes

**Fig. 1:**
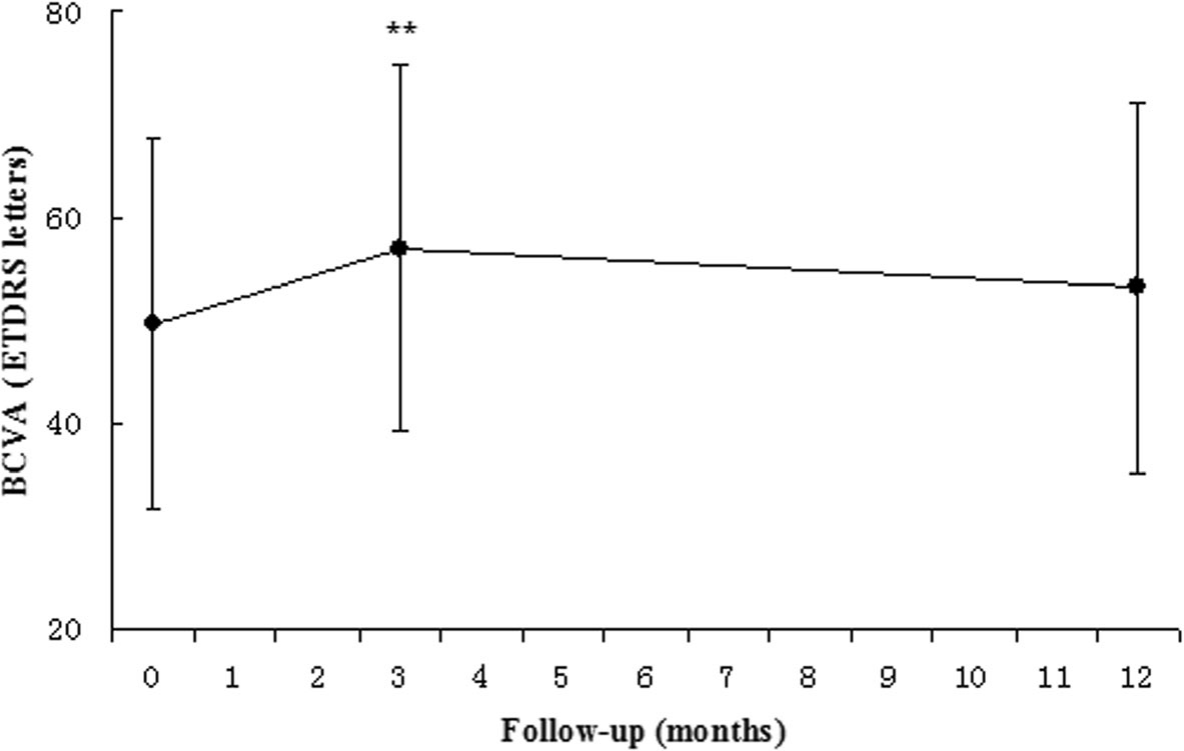
Mean BCVA of patients at the 3-month follow-up was 57.0 ± 17.7 letters, which was significantly increased by 7.3 ± 12.4 letters compared to baseline (*p* < 0.01). At the 12-month follow-up, the mean BCVA was 53.1 ± 18.0 letters, which was increased by 3.4 ± 15.4 letters compared to baseline, and there was no statistical difference (*p* > 0.05). Error bars indicate the upper and lower bounds of the 95% CIs. *Significant statistical difference (*p* < 0.05). **Significant statistical difference (*p* < 0.01) between groups

At the 3-month follow-up, which was 1 month after the initial three injections, the mean BCVA was 57.0 ± 17.7 letters. The mean BCVA was significantly increased by 7.3 ± 12.4 letters compared to baseline (*p* < 0.01). All 80 eyes showed stable vision (visual loss< 15 letters), and none of the patients experienced a visual acuity loss of more than 15 letters; the VA of 12 eyes (15.0%) gained more than 15 letters. Morphological changes in eyes at the 12-month follow-up are presented in Table 3. At the 12-month follow-up, the mean BCVA was 53.1 ± 18.0 letters. The mean BCVA was increased by 3.4 ± 15.4 letters compared to baseline, and there was no significant difference (*p* > 0.05, see Fig. 1). Of the 80 eyes, 78 eyes (97.5%) had stable vision; of these, 16 eyes (20.0%) gained more than 15 letters in terms of VA. Only 2 eyes (2.5%) suffered a severe visual loss corresponding to more than 15 letters. When 18 eyes that received PDT treatment (9 eyes with additional PDT and 9 eyes with combination treatment) were evaluated separately, the mean BCVA at the 12-month follow-up was 52.4 ± 20.0 letters. Therefore, the mean BCVA increased by 4.6 ± 19.9 letters. No significant difference was found compared to baseline for the 62 eyes that received ranibizumab monotherapy (*p* > 0.05).

**Table 3:** Changes measured by OCT and ICGA in eyes at the 12-month follow-up

Number of eyes (%)	Morphological changes
43 (60.6%)	Subretinal fluids absorbed
39 (55.7%)	PED resolved
15 (18.8%)	Complete regression of polyps
22 (27.5%)	Reduction in polyps

*OCT* optical coherence tomography, *ICGA* indocyanine green angiography, *PED* pigment epithelial detachment

### Changes in optical coherence tomography

**Fig. 2:**
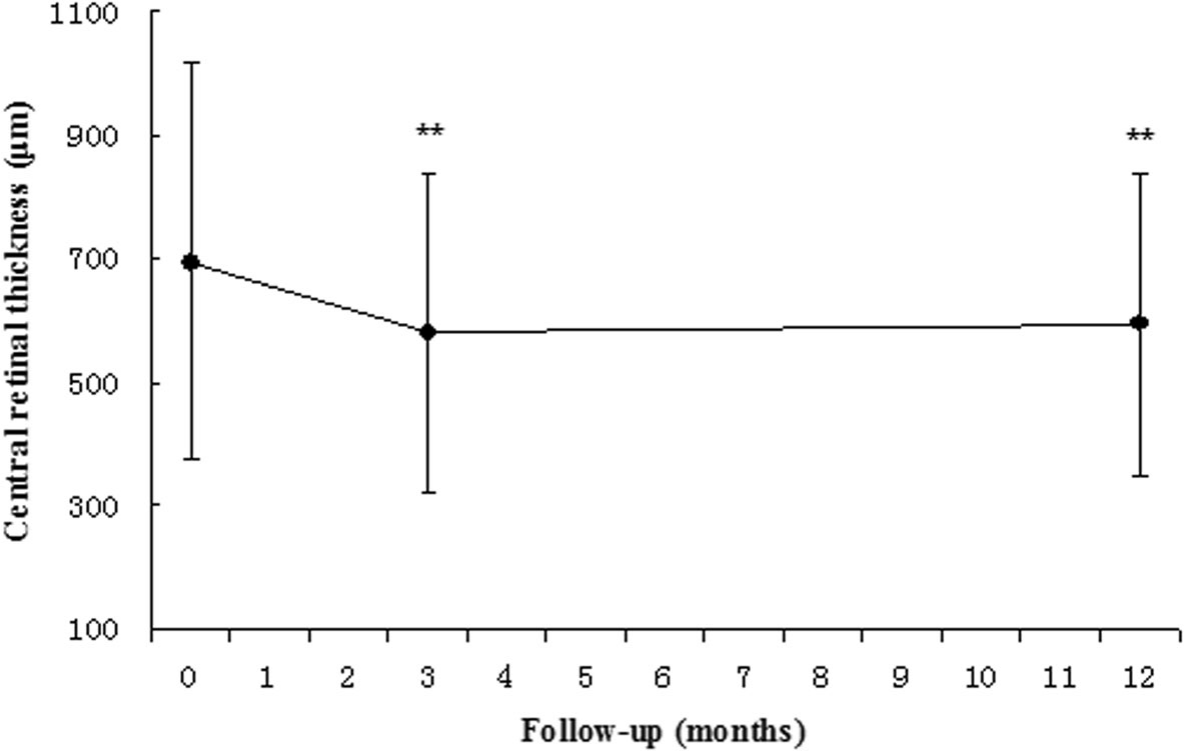
Mean CRT measured by OCT at the 3-month follow-up was 580.18 ± 257.04 μm, with an average decrease of 114.95 ± 237.21 μm compared to baseline, which represented a significant decrease (*p* < 0.01). At the 12-month follow-up, the mean CRT was 593.58 ± 243.64 μm. The mean decrease was 101.55 ± 256.07 μm compared to baseline (*p* < 0.01). Error bars indicate the upper and lower bounds of the 95% CIs. *Significant statistical difference (*p* < 0.05). **Significant statistical difference (*p* < 0.01) between groups

**Fig. 3:**
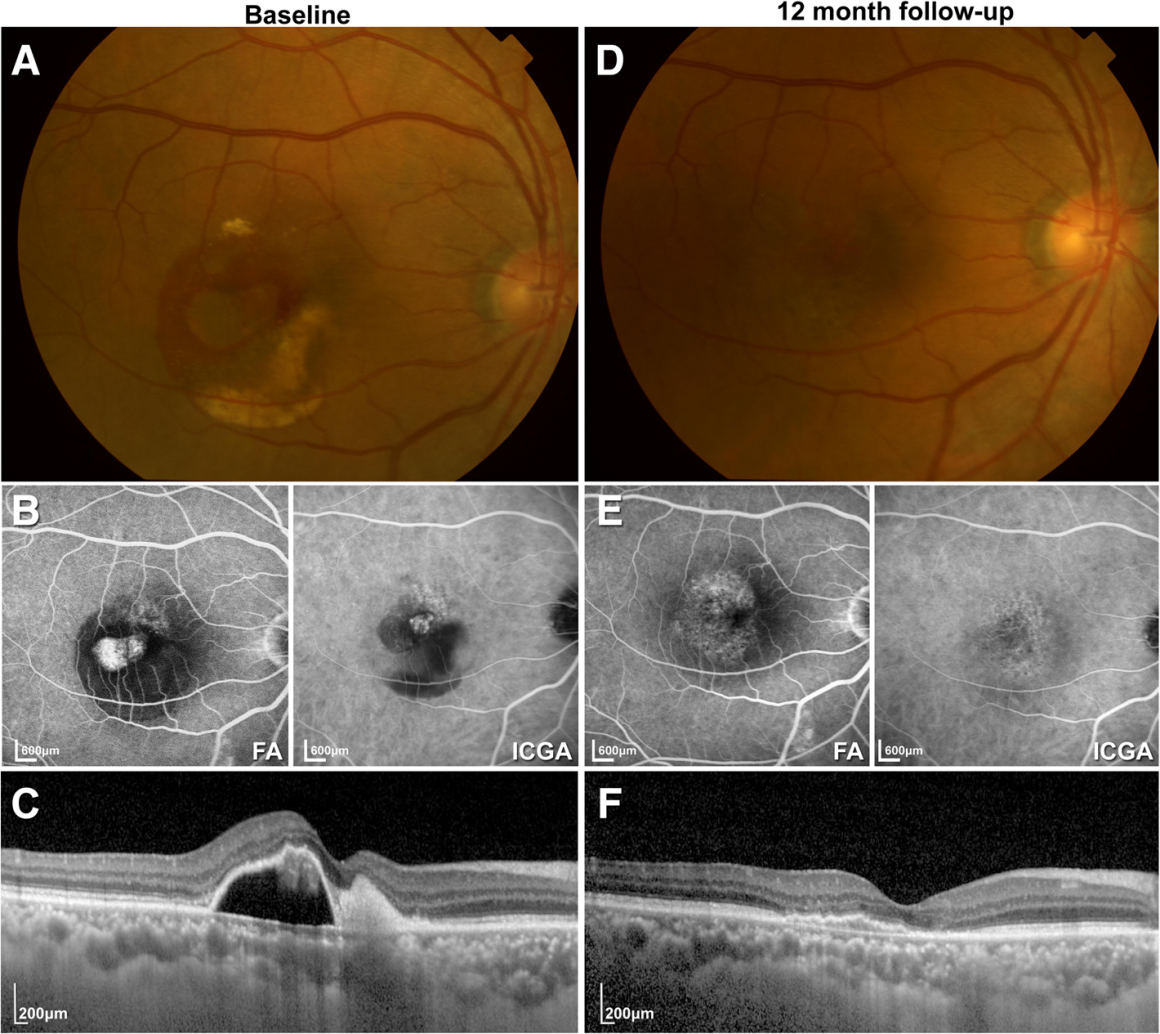
Case 14 presented with a visual acuity of 40 letters at baseline according to the ETDRS chart. The patient was treated with 5 consecutive intravitreal ranibizumab injections. At the 12-month follow-up, the visual acuity of the patient had improved to 63 letters according to ETDRS. **a** Fundus photography at baseline. **b** Baseline fluorescein angiography and indocyanine green angiography showing complex vascular and polypoidal lesions. **c** Optical coherence tomography at baseline. **d** Fundus photography at the 12-month follow-up. **e** Fluorescein angiography and indocyanine green angiography showing complete polyp regression at the 12 month follow-up. **f** Optical coherence tomography at the 12-month follow-up showing the resolution of PED and the return of the normal foveal depression

The mean CRT measured by OCT at the 3-month follow-up was 580.18 ± 257.04 μm, with an average decrease of 114.95 ± 237.21 μm compared to baseline, which demonstrated a significant decrease (*p* < 0.01). This difference was still present at the 12-month follow-up, at which time the mean CRT was 593.58 ± 243.64 μm. The mean decrease was 101.55 ± 256.07 μm compared to baseline (*p* < 0.01, see Fig. 2). In terms of morphology, at the 12-month follow-up, the subretinal fluids in 43 eyes (60.6%) were mostly or completely absorbed. Serous or hemorrhagic PED in 39 eyes (55.7%) was resolved (see Fig. 3e for an example). Morphological changes in eyes that received PDT at the 12-month follow-up are presented in Table 4. In the 18 eyes that received additional PDT treatment (9 eyes received additional PDT and 9 eyes received combination treatment), the reduction in the rates of the subretinal fluids and in PED were 56.3 and 53.3%, respectively.

**Table 4:** Changes measured by OCT and ICGA in eyes that received PDT at the 12-month follow-up

Number of eyes (%)	Morphological changes
9 (56.3%)	Subretinal fluids absorbed
8 (53.3%)	PED resolved
11 (61.6%)	Complete regression of polyps
3 (16.7%)	Reduction in polyps

*OCT* optical coherence tomography, *ICGA* indocyanine green angiography, *PDT* photodynamic therapy, *PED* pigment epithelial detachment

### Changes in indocyanine green angiography

To evaluate the degree of PCV, one of the most important measures was the regression rate of polyps. At the 12-month follow-up, we found that 15 eyes (18.8%) showed the complete regression of polyps and 22 eyes (27.5%) showed a reduction in polyps, indicating that a total of 37 eyes (46.3%) showed improvement regarding polyps (see Fig. 3b and Fig. 3d for examples). In the 18 eyes that received additional PDT treatment (9 eyes that received additional PDT and 9 eyes that received combination treatment), 11 eyes (61.1%) showed the complete regression of polyps, and 3 eyes (16.7%) showed a reduction in polyps. The polyp regression rate in eyes receiving PDT treatment was significantly higher than that in eyes treated with ranibizumab monotherapy.

### Factors predictive of the BCVA at final follow-up

Age, gender, baseline VA, baseline CRT, PED, retinal/subretinal hemorrhage, subretinal fluids, choroidal neovascularization (CNV), lesion location and greatest lesion diameter were considered possible predictive factors of BCVA at the 12-month follow-up. After simple linear regression analysis, the results showed that baseline VA, baseline CRT, retinal/subretinal hemorrhage, subretinal fluids and greatest lesion diameter were significantly correlated with relatively improved VA at the final follow-up (*p* < 0.01). Subsequently, stepwise multivariate linear regression analysis showed that baseline VA, subretinal fluids and greatest lesion diameter were significant and independent factors predictive of relatively improved VA at final follow-up (*p* < 0.01, see Table 5).

**Table 5:** Relationship between baseline characteristics and VA at the 12-month follow-up

Baseline Characteristic	*P* value of the univariate analysis of baseline characteristics and VA at the 12-month follow-up	*P* value of the multivariate analysis of baseline characteristics and VA at the 12-month follow-up
Age	0.24	
Gender	0.16	
Baseline VA	< 0.01	< 0.01
Baseline CRT	< 0.01	
PED	0.05	
Retinal/subretinal hemorrhage	< 0.01	
Subretinal fluids	< 0.01	< 0.01
CNV	0.91	
Lesion location	0.90	
Greatest lesion diameter	0.01	< 0.01

*VA* visual acuity, *CRT* central retinal thickness, *PED* pigment epithelial detachment, *CNV* choroidal neovascularization

### Adverse events

Topical adverse events such as elevated intraocular pressure, changes in lens opacity, vitreous hemorrhage, retinal detachment and endophthalmitis were not present among the patients. No patients required ocular surgery during follow-up. No systemic complications, such as thromboembolic events, were observed within 12 months of follow-up.

## Discussion

Because of the rapid progress of medical science, physicians have developed a deeper understanding of the pathogenesis of PCV. This has led to the development of a variety of treatment modalities, yet at present there is no consensus on the optimal treatment plan for PCV. PDT was once proposed as a standard treatment modality and is the most widely discussed modality in the literature. Recent PDT monotherapy studies have reported favorable results, with more than half of patients reporting the stabilization or improvement of vision and a polyp regression rate of 80–95% [21–25]. However, there are numerous disadvantages of PDT treatment, including its unsuitability for the treatment of multiple, widely-distributed lesions and its poor results for patients with substantial PED or submacular hemorrhage. Possible adverse events such as choroidal atrophy, massive subretinal or suprachoroidal hemorrhage and RPE tears after treatment also prevented its further use [26]. The latest development in PCV treatment was the use of anti-VEGF agents. Recent studies have found increased levels of VEGF in patients after PDT treatment, which may contribute to disease recurrence [27]. Various studies have found ranibizumab to be effective in reducing subretinal fluids and stabilizing or even improving visual acuity. Meanwhile, the complete regression of polyps was observed in only 14–40% of patients [12, 18, 28, 29]. This indicated that anti-VEGF therapy alone may have a relatively poor effect on the choroidal vascular branching network and polypoidal complexes. The EVEREST II study indicated that a combination therapy group had more improvement in VA and increased polyp regression rates compared to the ranibizumab monotherapy group at the 12-month follow-up [30]. Thus, physicians began to favor PDT and anti-VEGF combination therapy over PDT or anti-VEGF monotherapy alone. However, the current consensus for the treatment of PCV proposes that the ultimate objective is to stabilize visual acuity rather than reduce polyps. Therefore, more research data are needed to evaluate the visual outcomes of PCV patients initially treated with ranibizumab.

**Fig. 4:**
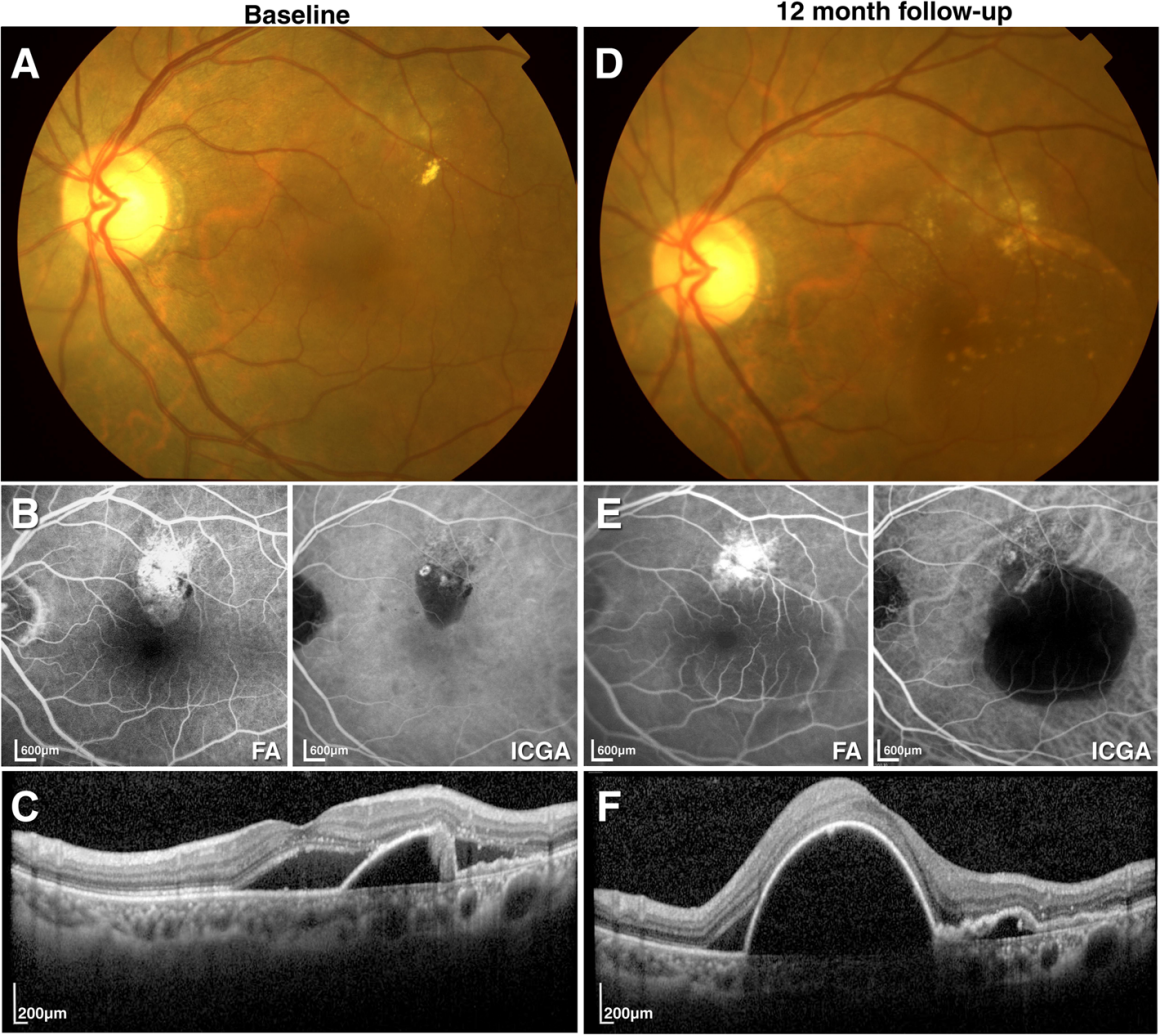
Case 46 presented with a visual acuity of 43 letters at baseline according to ETDRS. The patient was treated with 6 consecutive intravitreal ranibizumab injections. At the 12-month follow-up, the visual acuity of the patient was 35 letters according to ETDRS. **a** Fundus photography at baseline. **b** Fluorescein angiography and indocyanine green angiography at baseline showing polypoidal lesions. **c** Optical coherence tomography at baseline. **d** Fundus photography at the 12-month follow-up. **e** Fluorescein angiography and indocyanine green angiography at the 12-month follow-up. Note the presence of the polyp and the increased area of blocked fluorescence. **f** Optical coherence tomography at the 12-month follow-up showing PED recurrence

To observe the effect of intravitreal ranibizumab on treatment-naïve PCV, we enrolled 74 patients and collected data from 80 eyes, all of which received ranibizumab injections in a “3 + PRN” pattern. By the end of the study, 40/80 (50%) eyes had received more than 3 injections of only ranibizumab; 18/80 eyes (22.5%) were also subject to PDT treatment during follow-up. Our treatment strategy resulted in a statistically significant improvement in the BCVA and a reduction in CRT at the 3-month follow-up (*p* < 0.01). The mean BCVA improvement was 7.25 ± 12.44 letters, and the mean CRT reduction was 114.95 ± 237.21 μm. All eyes had stable vision, and none lost more than 15 letters in terms of VA. The short-term results of the intravitreal ranibizumab injections were satisfying; however, the 12-month follow-up results were less encouraging. There was still a significant reduction in CRT of 101.55 ± 256.07 μm (*p* < 0.01), but the mean BCVA was only slightly increased (3.40 ± 15.44 μm) and was not significantly different compared to baseline (*p* > 0.05). Two eyes (2.5%) suffered severe vision loss, one of which was due to choroidal atrophy after PDT treatment. Compared to the results of previous studies, the BCVA of the patients at the last follow-up did not improve significantly, although 78/80 eyes (97.5%) had stable vision [12, 28, 29, 31, 32]. This may be due to the retrospective nature of this study. Unlike randomized clinical trials, this study used a 3 + PRN treatment regimen, and the treatment decisions were based purely on the physician’s experience, which could result in under-treatment or delayed treatment and therefore lead to insignificant improvement in the BCVA at the end of the study. We believe that the visual results would have been improved if the retreatment criteria in clinical trials were followed in clinical practice. At the 12-month follow-up, the subretinal fluids in 43 eyes (60.6%) were mostly or completely absorbed, and PED in 39 eyes (55.7%) was resolved. The morphological changes shown during OCT were consistent with the results described by Kokame et al. [18]. Based on ICGA, 37 eyes (46.3%) had improvement in terms of polyps, but only 15 eyes (18.8%) showed complete regression (see Fig. 3b and Fig. 3d for examples of complete regression; see Fig. 4b and d for examples of recurrence). Of these 15 eyes, most had received PDT treatment after the 3 initial injections of ranibizumab, indicating that PDT played an important role in reducing polyps, although no significant differences were found in eyes that received ranibizumab monotherapy and PDT treatment in terms of BCVA, CRT, the reduction of subretinal fluids and PED. The EVEREST study showed that the combination therapy group had better VA and higher polyp regression rates compared to the ranibizumab monotherapy group [14, 30], while our study results demonstrated that ranibizumab given as an initial treatment could maintain VA in most PCV patients. Additional PDT treatment could improve polyp regression; however, no difference in VA was shown between the groups. The most recent study (PLANET study) with a large sample size, which compared anti-VEGF monotherapy and PDT rescue therapy, had similar results [20]. At the 12-month follow-up, more than 85% of patients treated with aflibercept monotherapy showed stabilized vision or functional improvement, and 40% of patients showed complete polyp regression. Of all the patients, 85% did not require PDT rescue treatment, indicating that aflibercept monotherapy could achieve VA stabilization.

In previous studies, there were discrepancies regarding the predictive factors of PCV visual outcomes. Possible predictive factors include age, gender, history of PDT, recurrence, baseline BCVA, baseline CRT, lesion size, lesion location, and the presence of characteristics such as grape-like polypoidal lesions, subretinal fluids, PED, CNV, lipid deposition and subretinal hemorrhage. Of these possible factors, decreased age, absence of a history of PDT, better baseline BCVA, smaller lesion size, and the absence of recurrence/PED/grape-like polypoidal lesions have been reported as independent factors correlated with better visual outcomes [31–34]. By using simple linear regression and stepwise multivariate linear regression, our study identified baseline BCVA, subretinal fluids and the greatest lesion diameter as independent factors predictive of relatively improved VA at final follow-up. A better baseline BCVA indicates that the disease was relatively mild; therefore, a therapeutic effect was guaranteed with timely treatment. The greatest lesion diameter was also repeatedly mentioned as a predictive factor in previous studies. It is easily understood that larger lesions are usually accompanied by choroidal neovascularization, massive hemorrhage or severe scarring, which can severely damage visual acuity. Subretinal fluids were a factor we identified that has not been previously mentioned in the literature. We believe that this is because eyes with subretinal fluids generally respond better to anti-VEGF treatment.

Further research is needed to determine which patients benefit more from ranibizumab monotherapy and which patients require additional PDT treatment. Several studies reported that eyes with relatively good baseline BCVA obtained increased benefit from ranibizumab treatment [28, 32, 33]. Other possible predictive factors include lesion type, lesion size, subretinal hemorrhage size, the presence of PED at diagnosis and a history of PDT [31, 32]. In our study, patients who underwent additional PDT treatment were often found to have large polyps and increased PED.

The main limitations of our study were its retrospective, nonrandomized nature and its relatively small sample size. Lesions were not classified, and the decision to administer additional treatment was based purely on the physician’s experience. All of these factors could result in selection bias. In addition, not all patients underwent ICGA at the 3-month follow-up; therefore, polyp regression at the 12-month follow-up could possibly be the result of the natural course of disease. The follow-up period was also relatively short. PCV is a disease with possible recurrence; therefore, longer follow-up periods are required. Because of the age distribution in the PCV disease population, cataracts are a common condition in patients. Although significant changes in lens opacity were not observed during follow-up, enrolling patients both with and without cataract extraction in the study population is a potential source of heterogeneity.

## Conclusion

Our findings demonstrated that three monthly injections of ranibizumab as an initial treatment could significantly improve VA in PCV patients in the short term. At 12 months postinjection, ranibizumab treatment could stabilize VA in most PCV patients. Patients who received additional PDT treatment had a significantly increased polyp regression rate. Further research is needed to determine the optimal type of patient who should receive ranibizumab monotherapy.

## Data Availability

The datasets used and/or analyzed during the current study are available from the corresponding author upon reasonable request.

## Abbreviations

AMD: Age-related macular degeneration
BCVA: Best corrected visual acuity
BVN: Branching vascular network
CNV: Choroidal neovascularization
CRT: Central retinal thickness
ETDRS: Early treatment diabetic retinopathy study
FA: Fluorescein angiography
ICGA: Indocyanine green angiography
IOP: Intraocular pressure
OCT: Optical coherence tomography
PCV: Polypoidal choroidal vasculopathy
PDT: Photodynamic therapy
PED: Pigment epithelial detachment
RPE: Retinal pigment epithelium
VEGF: Vascular endothelial growth factor
